# Annexin A1 Attenuates Neutrophil Migration and IL-6 Expression through Fpr2 in a Mouse Model of Streptococcus suis-Induced Meningitis

**DOI:** 10.1128/IAI.00680-20

**Published:** 2021-02-16

**Authors:** Chengpei Ni, Song Gao, Yuling Zheng, Peng Liu, Yajie Zhai, Wenhua Huang, Hua Jiang, Qingyu Lv, Decong Kong, Yongqiang Jiang

**Affiliations:** a State Key Laboratory of Pathogen and Biosecurity, Institute of Microbiology and Epidemiology, Academy of Military Medical Sciences, Beijing, China; b Department of Central Laboratory, Liaocheng People's Hospital, Liaocheng, Shandong Province, China; c The Sixth Medical Center of Chinese PLA General Hospital, Beijing, China; Washington State University

**Keywords:** *Streptococcus suis*, meningitis, formyl peptide receptor 2, annexin A1

## Abstract

Streptococcus suis serotype 2 is a crucial pathogenic cause of bacterial meningitis, a life-threatening disease with neurological sequelae and high rates of mortality. Inflammation triggered by S. suis infection must be precisely regulated to prevent further tissue damage. As a glucocorticoid anti-inflammatory mediator, annexin A1 (AnxA1) mainly acts through formyl peptide receptor 2 (Fpr2) to alleviate inflammation in the peripheral system. In this study, we evaluated the roles of AnxA1 and Fpr2 in a mouse model of S. suis meningitis created via intracisternal infection in Fpr2-deficient (Fpr2^−/−^) and wild-type (WT) mice. We revealed that Fpr2^−/−^ mice were highly susceptible to S. suis meningitis, displaying increased inflammatory cytokine levels, bacterial dissemination, and neutrophil migration compared with WT mice. Additionally, AnxA1 exerted anti-inflammatory effects through Fpr2, such as attenuation of leukocyte infiltration, inflammatory mediator production, and astrocyte or microglial activation in the brain. Importantly, we found that the antimigratory function of AnxA1 decreases neutrophil adherence to the endothelium through Fpr2. Finally, an *in vitro* study revealed that AnxA1 potentially suppresses interleukin-6 (IL-6) expression through the Fpr2/p38/COX-2 pathway. These data demonstrated that Fpr2 is an anti-inflammatory receptor that regulates neutrophil migration in mice with S. suis meningitis and identified AnxA1 as a potential therapeutic option.

## Abstract (precis)

Streptococcus suis serotype 2 is a crucial pathogenic cause of bacterial meningitis, a life-threatening disease with neurological sequelae and high rates of mortality. Inflammation triggered by S. suis infection must be precisely regulated to prevent further tissue damage.

## INTRODUCTION

Streptococcus suis is a crucial swine and human pathogen responsible for sudden death, septic shock, and meningitis, which are characterized by exacerbated inflammation in both systemic and central nervous system (CNS) infections (1, 2). Meningitis is the most common presentation of this infection in both Europe and Asia, and sepsis is the second most common S. suis-related zoonosis (3). Of the different serotypes described according to the manifestation of the capsular polysaccharide or its respective genes, serotype 2 is often associated with lethal infections (4). However, no effective vaccine is available to control infections caused by this bacterium, which necessitates new therapeutic strategies.

The complexity of S. suis-induced disease is attributable to multiple local and systemic immune reactions involving the production of inflammatory mediators, such as cell stress markers, bioactive lipid mediators, and cytokines, which result in multiple-organ damage and ultimately death (5). Drastic and persistent inflammatory responses may result in tissue injury as a by-effect of the intense induction of reactive oxygen species, neutrophil extracellular traps (NETs), and proteases by neutrophils (6). Therefore, inflammation should be precisely regulated to guarantee the ultimate resolution of infection and remodeling of homeostasis. Different from anti-inflammatory mediators, proresolution mediators reduce inflammation without compromising host defenses against pathogens.

Annexin A1 (AnxA1) is a 37-kDa calcium-dependent phospholipid-binding protein known for its anti-inflammatory effects and proresolution (7). Several pharmacologic investigations revealed that AnxA1 inhibits neutrophil extravasation in models of acute and chronic inflammation, as well as in models of systemic inflammation (8). AnxA1 is an effective inhibitor of leukocyte infiltration that influences the detachment of leukocyte from the postcapillary endothelium in different kinds of vascular beds with inflammation, including the mesentery and brain (9, 10). The protein additionally modulates the induction of proinflammatory mediators, including those derived from the activation of PLA2, cyclooxygenase-2 (COX-2), and inducible nitric oxide synthase (11–13).

AnxA1 principally acts through formyl peptide receptor 2 (Fpr2), a G protein-coupled receptor that can discover the existence of bacteria and function as a chemotactic receptor (14, 15). There are six members of the formyl peptide receptor gene family in murine species and three in humans. Murine Fpr2 is a receptor that is most similar to human Fpr2 (16, 17). Fpr2 transduces the anti-inflammatory effects of AnxA1 in a variety of systems and neuroprotective effects in humans, and it can also mediate proinflammatory reaction to serum amyloid A (SSA) and other proinflammatory peptides (13). It was reported that the development and resolution of inflammation involve both proinflammatory and anti-inflammatory biochemical mechanisms, ultimately facilitating the restoration of homeostasis in inflamed tissues (18). Although the protective effects of the AnxA1-Fpr2 system have been well studied in a variety of disease models, only a few studies have concentrated on the functions of this system in bacterial infection (19). Recent studies demonstrated that the AnxA1-Fpr2 interaction regulates the inflammatory reaction and bacterial dissemination in a mouse model of experimental pneumococcal pneumonia (20). However, the specific role of the AnxA1-Fpr2 pathway in S. suis meningitis remains to be determined.

In this study, we used Fpr2^−/−^ mice to estimate the role of murine Fpr2 and exogenous AnxA1 in S. suis-induced meningitis. With a particular emphasis on the function of AnxA1 in the regulation of neutrophil recruitment and interleukin-6 (IL-6) production in S. suis-induced meningitis, we demonstrate an immunomodulatory role for AnxA1 in S. suis-induced infection through Fpr2. AnxA1 conferred protection against inflammatory responses and neutrophil invasion during S. suis-induced meningitis mainly through Fpr2. We report for the first time evidence that the anti-inflammatory effects of AnxA1 in mice with S. suis meningitis are mediated by Fpr2, thereby providing a new therapeutic target for S. suis meningitis treatment.

## RESULTS

### Fpr2 deficiency aggravates the host response to S. suis meningitis.

**FIG 1:**
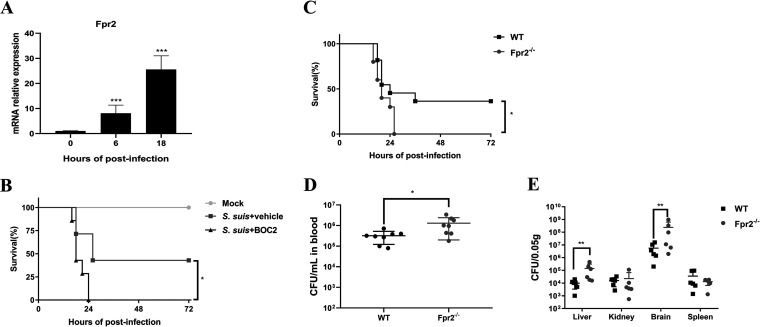
Fpr2 deficiency aggravates meningitis resulting from severe S. suis infection. (A) Wild-type (WT) mice were intracisternally inoculated with 1.25 × 10^5^ CFU of S. suis 05ZYH33, and total RNA was isolated from the brain at the indicated time points. Fpr2 mRNA levels were evaluated using quantitative real-time PCR. The results are presented relative to GADPH mRNA amplification. (B) Survival curves of mice treated with BOC-2 or vehicle. WT mice were treated with BOC-2 (600 ng/kg) or vehicle for 1 h before S. suis 05ZYH33 (1.25 × 10^5^ CFU) infection. The survival rates of the two groups were compared using the log rank test (*n* = 7/group). (C) WT and Fpr2^−/−^ mice were intracisternally inoculated with S. suis 05ZYH33 (1.25 × 10^5^ CFU). The survival rates of the two groups were compared using the log rank test (*n* = 10/group), and bacterial loads in the (D) blood or (E) tissue homogenate of the liver, kidney, brain, and spleen at 14 h were determined via colony plate counting. *, *P* < 0.05; **, *P* < 0.01; ***, *P* < 0.001.

An initial experiment was conducted to evaluate whether Fpr2 was involved in S. suis infection. Fpr2 transcription was investigated in mice intracisternally infected with 1.25 × 10^5^ CFU of the S. suis 05ZYH33 strain for 0, 6, or 18 h. The infection induced significant Fpr2 transcription in the brain after 6 and 18 h (Fig. 1A). Moreover, treatment with the Fpr2 pan-antagonist BOC-2 more strongly increased the susceptibility to S. suis infection than vehicle (lethality rate of 100% versus 57.4% [Fig. 1B]). These results indicated the protective function of Fpr2 during S. suis meningitis.

To determine the role of Fpr2 in the development of S. suis meningitis, wild-type (WT) and Fpr2^−/−^ mice were intracisternally inoculated with S. suis 05ZYH33 (1.25 × 10^5^ CFU), and lethality rates and bacterial loads were evaluated. Fpr2^−/−^ mice were more susceptible to S. suis infection than WT mice (lethality rate of 100% versus 60% [Fig. 1C]). The bacterial load was determined via colony plate counting after 14 h of infection, and significant differences were observed in blood, liver, and brain bacterial counts between Fpr2^−/−^ and WT mice (Fig. 1D and E). Fpr2 deficiency aggravated meningitis resulting from severe S. suis infection.

**FIG 2:**
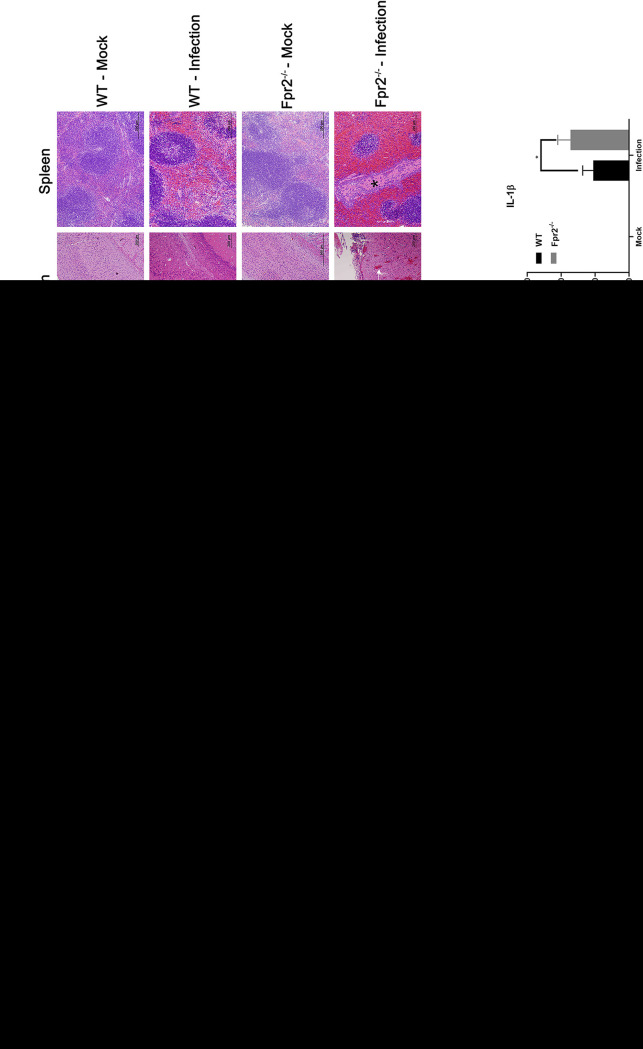
Fpr2 deficiency increases inflammatory responses and histopathological lesions in S. suis meningitis. Wild-type (WT) and Fpr2^−/−^ mice were intracisternally inoculated with S. suis 05ZYH33 (1.25 × 10^5^ CFU). (A to F) The transcriptional levels and (G to J) production amounts of proinflammatory mediators in the brains of mice at 14 h after infection are shown. (K) Hematoxylin and eosin staining of infected tissue sections after 14 h of infection. The asterisk indicates necrosis, white arrows indicate intracerebral hemorrhage, and black arrows indicate thickening of the meninges. The horizontal line indicates 200 μm. *, *P* < 0.05; **, *P* < 0.01; ***, *P* < 0.001.

To assess the regulatory role of Fpr2 in inflammation induced by S. suis infection, we measured the levels of inflammatory mediators by quantitative real-time PCR and enzyme-linked immunosorbent assay (ELISA) and conducted histopathological studies at 14 h after infection in WT and Fpr2^−/−^ mice. Compared with the findings in WT mice, Fpr2^−/−^ mice exhibited higher levels of proinflammatory mediators during both transcription (Fig. 2A to F) and production (Fig. 2G to J), especially CXCL2, IL-6, and IL-1β (production amounts of tumor necrosis factor alpha [TNF-α] and gamma interferon [IFN-γ] were below the detection levels [data not shown]) and showed aggravation of pathological injury in multiple tissues (Fig. 2K).

**FIG 3:**
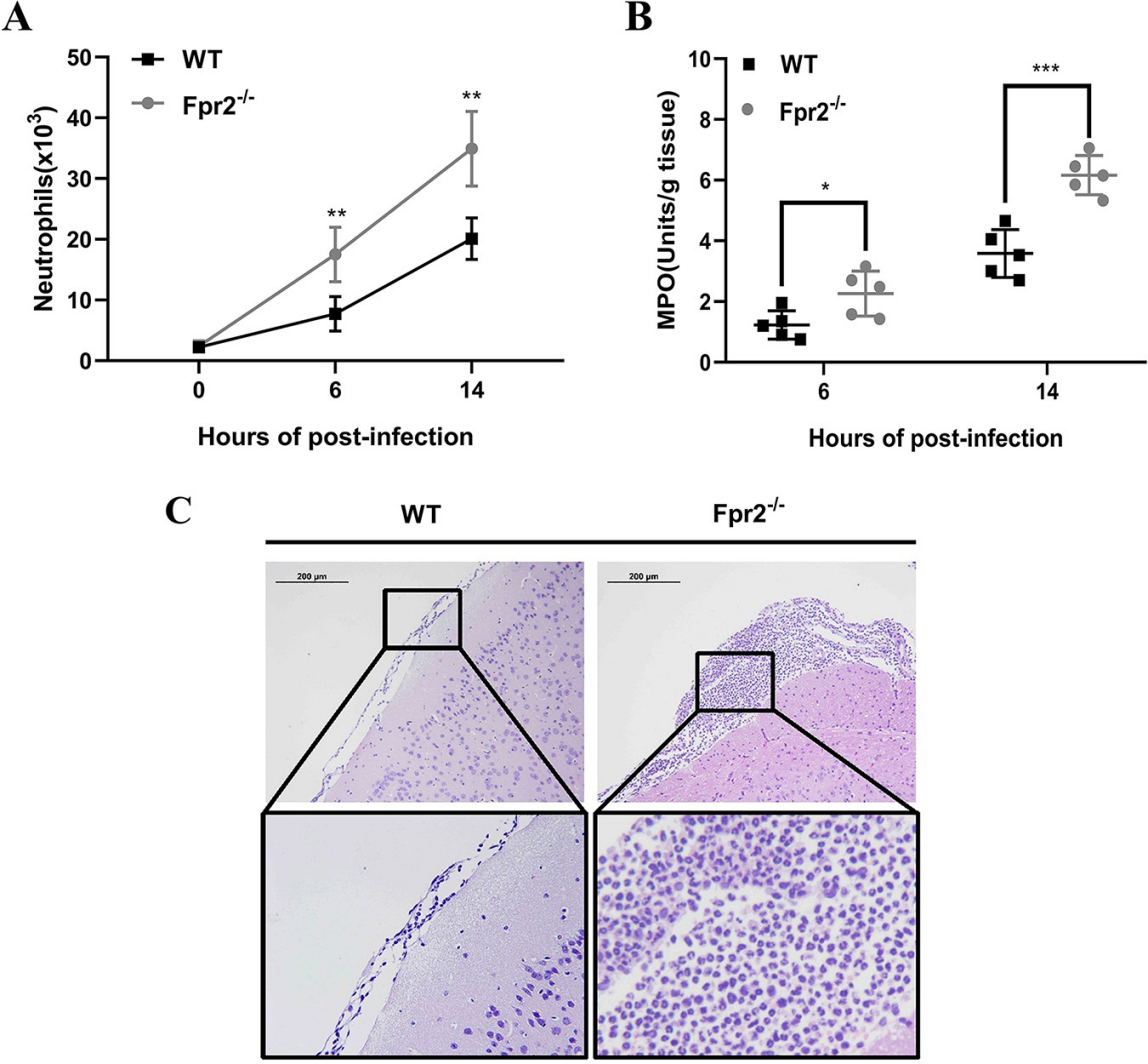
Absence of Fpr2 increases neutrophil recruitment in S. suis meningitis. Wild-type (WT) and Fpr2^−/−^ mice were intracisternally inoculated with S. suis 05ZYH33 (1.25 × 10^5^ CFU). (A) Neutrophil counts in the brains of WT and Fpr2^−/−^ mice at the indicated times after infection. (B) Myeloperoxidase (MPO) activity in the brains of WT and Fpr2^−/−^ mice at the indicated time points. (C) Hematoxylin and eosin staining of infected brain sections from WT and Fpr2^−/−^ mice at 14 h after infection. *, *P* < 0.05; **, *P* < 0.01; ***, *P* < 0.001.

Because neutrophils are crucial mediators of host defenses during acute bacterial meningitis (21), neutrophil counts and myeloperoxidase (MPO) activity (an indirect indicator of neutrophil recruitment) in the brain after infection were assessed. Fpr2 deficiency increased neutrophil recruitment in the brain at 6 and 14 h after infection (Fig. 3A). Consistent with the enhancement of neutrophil infiltration, Fpr2^−/−^ mice also exhibited higher brain MPO activity than WT mice (Fig. 3B). The augmentation of neutrophil recruitment following S. suis meningitis in Fpr2^−/−^ mice was also reflected in brain slices (Fig. 3C).

Collectively, these data indicated that Fpr2 plays a potentially critical role in controlling inflammatory responses and neutrophil migration in S. suis meningitis.

### AnxA1 reduces neutrophil invasion and bacterial loads through Fpr2 in mice with S. suis meningitis.

AnxA1 in the CNS participates in anti-inflammatory effects and the maintenance of brain stability, and the biological effects of AnxA1 and its mimetic peptides are mediated by Fpr2 (22, 23). S. suis infection increased intracellular AnxA1 cleavage and exocytosis, which was evidenced by increased AnxA1 levels in the supernatant of S. suis-infected bEnd.3 cells (a mouse brain-derived endothelial cell line) and brain tissue from infected mice (see Fig. S1A to D in the supplemental material). Although no significant difference was observed between the two genotypes of mice in terms of endogenous AnxA1 expression after infection, the question was whether the anti-inflammatory effects of AnxA1 were restricted with Fpr2 deficiency.

**FIG 4:**
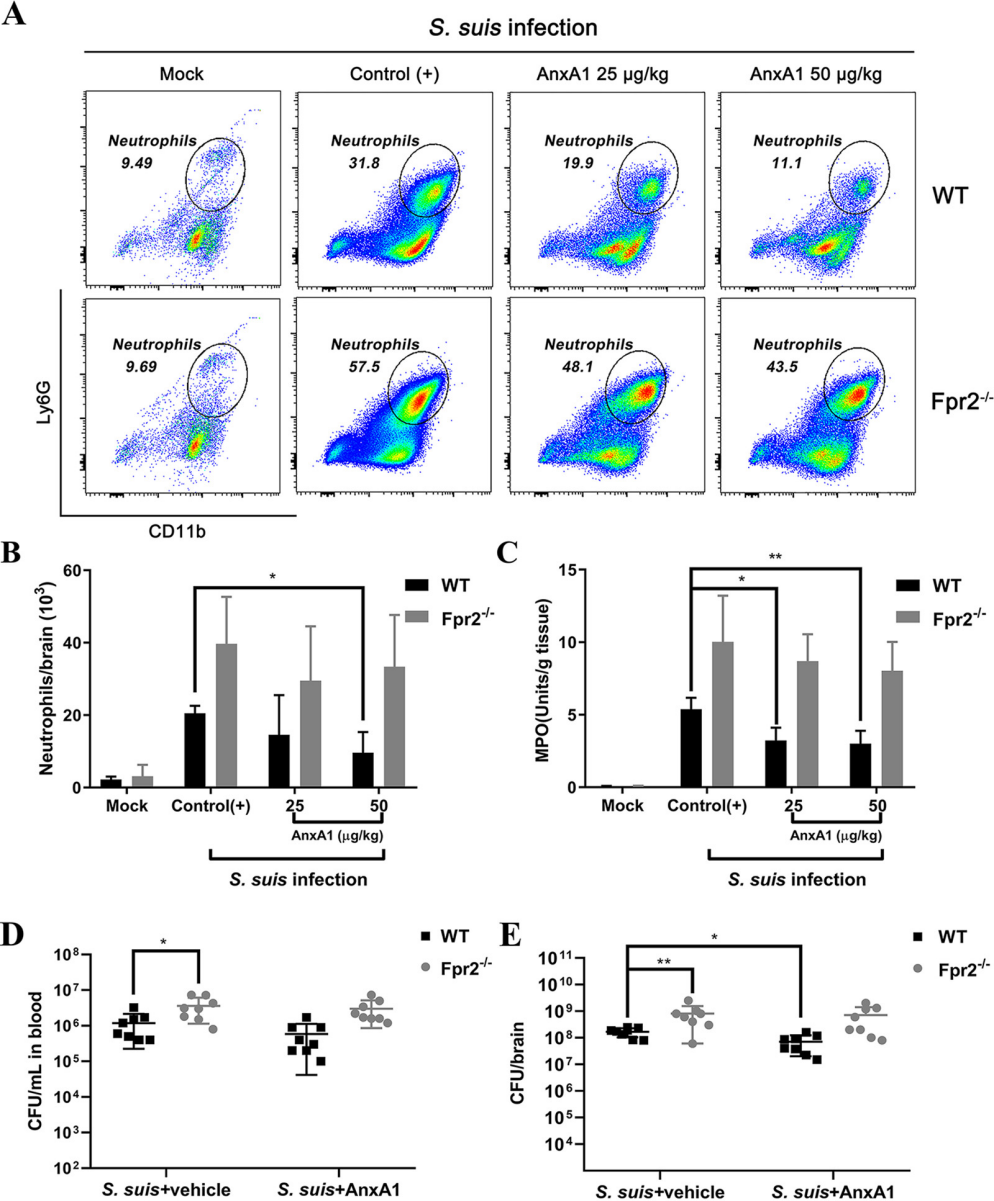
AnxA1 reduces bacterial loads and neutrophil invasion during S. suis meningitis in wild-type (WT) mice but not Fpr2^−/−^ mice. AnxA1 (25 or 50 µg/kg) or vehicle [Control(+)] was administered intravenously prior to the intracisternal inoculation of S. suis 05ZYH33 (1.25 × 10^5^ CFU) in WT and Fpr2^−/−^ mice. At 14 h after infection, (A) the proportion and (B) counts of Ly6G^+^ CD11b^+^ neutrophils among leukocytes (CD45^+^) were quantified via flow cytometry and cell counter. (C) The MPO activity of brain was also evaluated. AnxA1 (50 µg/kg) or vehicle was administered intravenously prior to the intracisternal inoculation of S. suis (1.25 × 10^5^ CFU) in WT and Fpr2^−/−^ mice, then bacterial loads in (D) blood and (E) brain at 14 h after infection were determined via colony plate counting. *, *P* < 0.5; **, *P* < 0.01.

In order to examine whether exogenous AnxA1 treatment could modulate cellular influx to the brain through Fpr2 in mice with S. suis meningitis, recombinant AnxA1 protein was used in the following experiments. Before S. suis (1.25 × 10^5^ CFU) infection, a prophylactic dose of AnxA1 (25 or 50 µg/kg of body weight) was given to WT and Fpr2^−/−^ mice via the intravenous (i.v.) route, after which neutrophil counts and MPO levels were measured. Analyses of brain tissue via flow cytometry revealed that leukocyte activation was reversed by treatment with 50 µg/kg AnxA1 in WT mice (Fig. 4A and B). Exogenous AnxA1 exhibited potent antimigratory effects in WT mice in a dose-dependent manner. Specifically, 50 µg/kg AnxA1 significantly reduced Ly6G^+^ CD11b^+^ neutrophil cell infiltration in the brains of S. suis-infected WT mice compared with the findings in WT control [control(+)] mice (46%, *P* < 0.05 [Fig. 4B]). However, cell infiltration was not significantly affected by AnxA1 treatment in Fpr2^−/−^ mice. This finding was consistent with the observation that AnxA1 (50 µg/kg) significantly decreased MPO expression only in WT mice (Fig. 4C). These results demonstrated the antimigratory function of AnxA1 was mediated by Fpr2 in mice with S. suis meningitis.

S. suis infection in the intracerebral region has been inferred to induce a local proinflammatory response (24). Thus, the bacterial loads in the blood and brain homogenate of infected mice treated with 50 µg/kg AnxA1 or vehicle were assessed at 14 h after infection. AnxA1 treatment did not influence blood bacterial loads in either WT or Fpr2^−/−^ mice (Fig. 4D). Conversely, AnxA1-treated WT mice exhibited lower brain bacterial loads than their vehicle-treated counterparts, but this finding was not replicated in Fpr2^−/−^ mice (Fig. 4E). Together, these data demonstrated that AnxA1 suppresses neutrophil invasion and promotes bacterial removal in the brains of mice with S. suis-induced meningitis through Fpr2.

### AnxA1 attenuates inflammatory responses and brain damage through Fpr2 in mice with S. suis infection meningitis.

**FIG 5:**
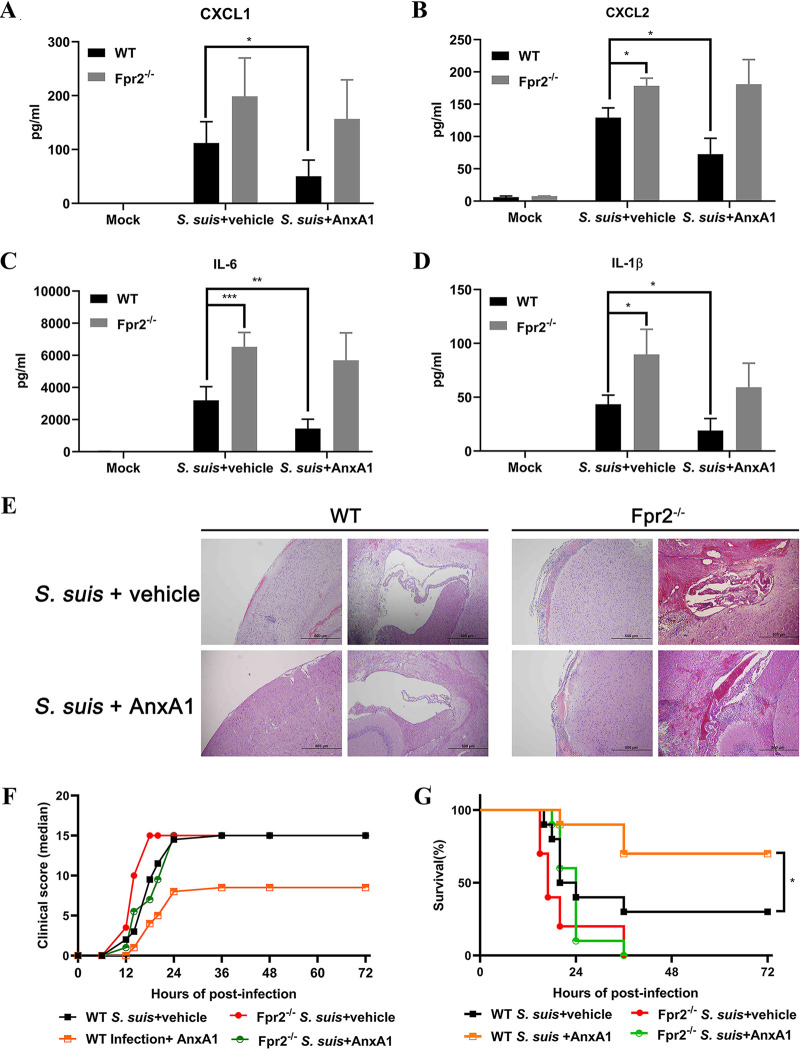
AnxA1 prevented S. suis-induced increases in inflammatory responses and brain damage in wild-type (WT) but not Fpr2^−/−^ mice. AnxA1 (50 µg/kg) or vehicle was administered intravenously prior to the intracisternal inoculation of S. suis 05ZYH33 (1.25 × 10^5^ CFU) in WT and Fpr2^−/−^ mice. (A to D) Brain proinflammatory mediator levels in WT and Fpr2^−/−^ mice treated with AnxA1 or vehicle at 14 h after S. suis infection were determined using ELISA. (E) Hematoxylin and eosin staining of infected brain sections was performed after 14 h of infection. The size bars represent 500 μm. (F) Clinical score and (G) survival curves of WT and Fpr2^−/−^ mice after S. suis infection are shown. The survival rates were compared using the log rank test (*n* = 10/group). *, *P* < 0.05; **, *P* < 0.01; ***, *P* < 0.001.

The next experiments focused on the role of AnxA1 in proinflammatory cytokine production and brain injury after S. suis infection. We treated WT and Fpr2^−/−^ mice with AnxA1 (50 µg/kg) or vehicle and analyzed brain cytokine levels and histopathological change at 14 h after S. suis infection (1.25 × 10^5^ CFU). ELISA confirmed that the increased cytokine production (CXCL1, CXCL2, IL-6, and IL-1β) induced by bacterial infection was reverted by AnxA1 treatment in WT mice at 14 h after infection (Fig. 5A to D). Although Fpr2^−/−^ mice appeared to have a higher level of CXCL1 after infection, there was no significant difference between the two genotypes of mice. The result was consistent with previous findings (Fig. 2G). Notably, IL-6 levels were significantly (*P* < 0.01) reduced after AnxA1 treatment in WT but not Fpr2^−/−^ mice, which suggests that the high levels of IL-6 demonstrate it is the primary cytokine responsible for local proinflammatory activity during the early stages of S. suis meningitis (Fig. 5C). Histopathological analysis of brain tissues from WT and Fpr2^−/−^ mice revealed that AnxA1 administration prevented infection-induced brain damage in WT mice. AnxA1 treatment restored most of the WT phenotype, including the amelioration of intracerebral hemorrhage and granulocyte infiltration, whereas this effect was not observed in Fpr2^−/−^ mice (Fig. 5E). Clinical score and survival experiments were also conducted. In the absence of AnxA1 treatment, most infected mice regardless of genotype presented with severe clinical signs and died (more than 60%) within 2 days, whereas AnxA1 administration significantly alleviated severe clinical signs and prolonged survival in WT mice but not Fpr2^−/−^ mice (Fig. 5F and G). The survival curves revealed significantly decreased mortality rates in AnxA1-treated WT mice compared to their control counterparts (lethality of 30% versus 70% [Fig. 5G]).

Collectively, these data indicated that Fpr2 mediates the anti-inflammatory activity of AnxA1 *in vivo* during S. suis meningitis, thereby protecting mice from severe local inflammatory responses and brain damage. Fpr2 deficiency was associated with insensitivity to the anti-inflammatory effects of AnxA1.

### AnxA1 attenuates astrocyte and microglial activation through Fpr2 in S. suis meningitis.

**FIG 6:**
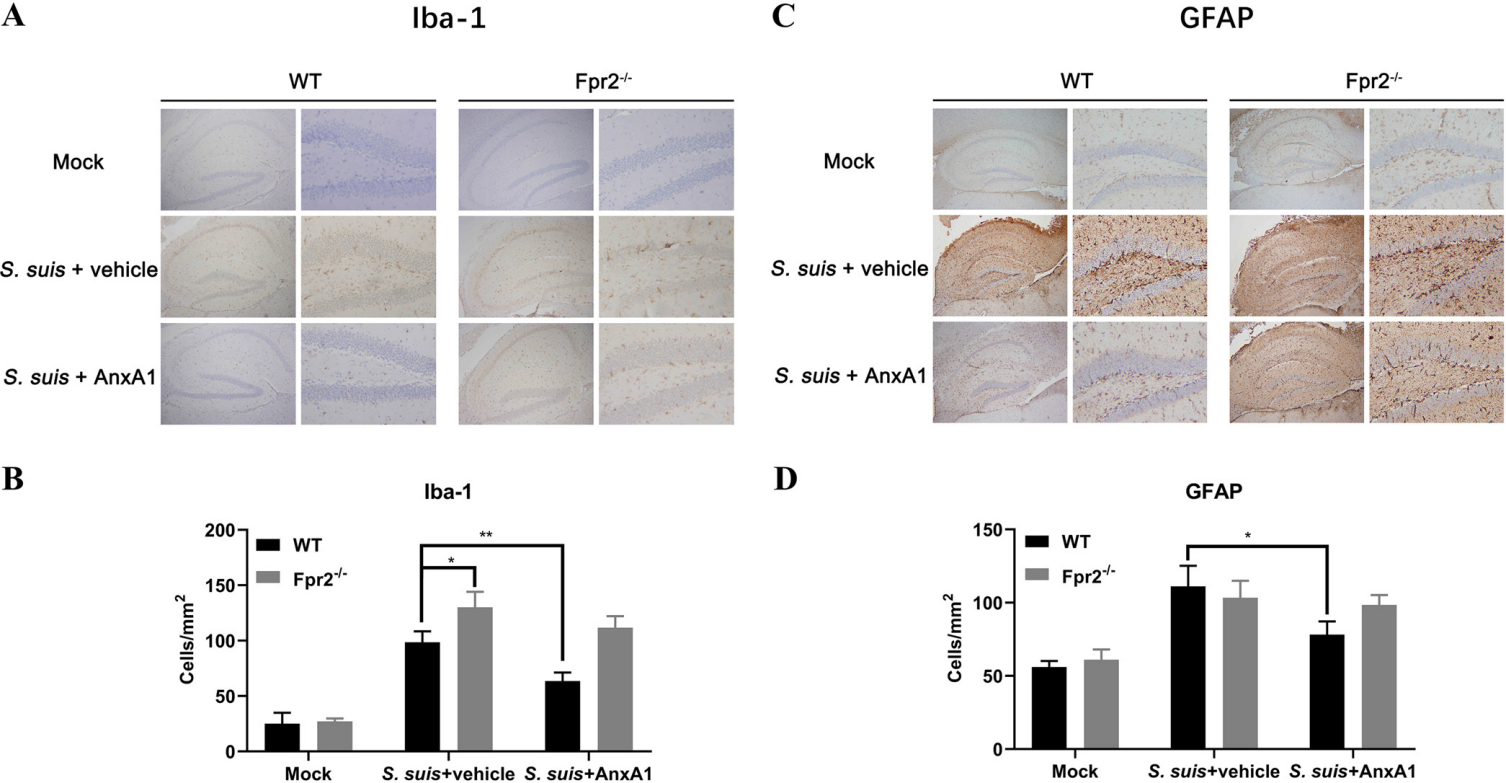
AnxA1 prevents S. suis infection-induced increases in astrocyte and microglial density in wild-type (WT) but not Fpr2^−/−^ mice. AnxA1 (50 µg/kg) or vehicle was administered intravenously prior to the intracisternal inoculation of S. suis 05ZYH33 (1.25 × 10^5^ CFU) in WT and Fpr2^−/−^ mice. Brain sections were stained with (A) anti-Iba-1 or (C) anti-GFAP to identify activated microglial cells and astrocytes at 14 h after infection. The density of (B) Iba-1- or (D) GFAP-expressing cells was calculated per square millimeter of the hippocampus. *, *P* < 0.05; **, *P* < 0.01.

The activation of astrocytes and microglia result in the massive production of chemokines and cytokines followed by neutrophil recruitment during bacterial meningitis (25). The following set of experiments estimated whether the AnxA1 plays a role in astrocyte and microglial activation in mice with S. suis meningitis. Following AnxA1 (50 µg/kg) or vehicle administration (i.v.) prior to S. suis intracisternal inoculation in WT and Fpr2^−/−^ mice, the astrocyte or microglial density at 14 h after infection was determined via immunohistochemistry using antibodies targeting the astrocyte marker GFAP and microglia marker Iba-1. The assessments were performed in the hippocampus as a clearly definable brain region involved in the pathogenesis of bacterial meningitis (26). At 14 h after infection, the densities of GFAP- and Iba-1-expressing cells were increased in both genotypes compared with the findings in the mock-infected controls. Although the density of Iba-1-expressing cells was higher in Fpr2^−/−^ mice with meningitis than in WT S. suis-infected mice, AnxA1 reduced microglial activation only in WT mice (Fig. 6A and B). Concerning astrocyte activation, AnxA1 treatment effectively reduced the number of GFAP-positive astrocytes compared with the findings in untreated infected animals, but this treatment was ineffective in Fpr2^−/−^ mice (Fig. 6C and D).

In sum, these results suggested that AnxA1 reduces astrocytic and microglial activation in mice with S. suis meningitis, and these effects were mediated by Fpr2.

### AnxA1 decreases neutrophil adhesion through Fpr2.

**FIG 7:**
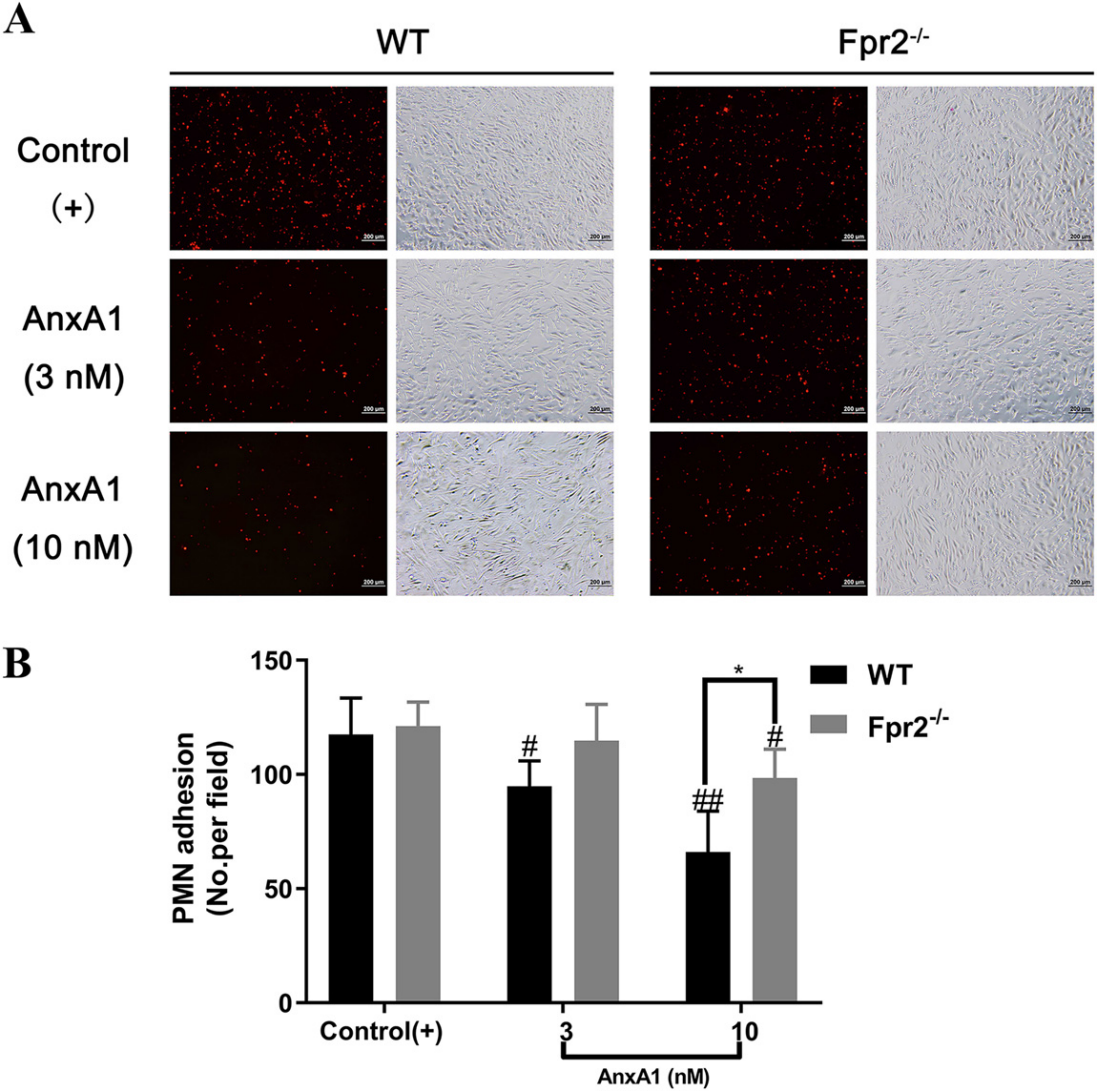
AnxA1 decreases neutrophil adhesion through Fpr2. AnxA1 (3 or 10 nM) was administered to wild-type (WT) and Fpr2^−/−^ neutrophils 10 min prior to the addition of HkSs-stimulated bEnd.3 cells. (A) Representative image of neutrophil adhesion to bEnd.3 cells. The space bars represent 200 μm. (B) The adhesive cell count was normalized to that of the non-AnxA1 treatment control. #/*, *P* < 0.05, and ##, *P* < 0.01, compared with the control(+) group (#) in the same mouse strain or as indicated (*).

It has been reported that AnxA1 controls neutrophil adhesion to the activated postcapillary endothelium of inflamed vascular beds (27). The activation of bEnd.3 cells by heat-killed S. suis (HkSs) treatment would be proven by increased expression of adhesion-related proteins ICAM-1 and IL-6 (see Fig. S2A and B in the supplemental material). Accordingly, we examined the comparative effect of AnxA1 on the adhesion of WT or Fpr2^−/−^ neutrophils to bEnd.3 cells in response to HkSs. AnxA1 (3 or 10 nM) was administered 10 min prior to the addition of WT or Fpr2^−/−^ neutrophils to bEnd.3 cells stimulated with HkSs. The result revealed that AnxA1 significantly decreased the adherence of WT neutrophils to bEnd.3 cells, especially in the 10 nM AnxA1 treatment group (Fig. 7A and B). Fpr2 deficiency was associated with insensitivity to the deadhesion effect of AnxA1, as a concentration of 10 nM was required for deadhesion in Fpr2^−/−^ neutrophils. Notably, AnxA1-treated neutrophils exhibited lower adhesive ability than control cells irrespective of genotype, which indicated that other receptors also participate in AnxA1-mediated neutrophil deadhesion. These results suggested that AnxA1 decreases neutrophil adhesion to the activated endothelium mainly through Fpr2.

### AnxA1 decreased the effects of IL-6 through the Fpr2/p38/COX-2 pathway.

**FIG 8:**
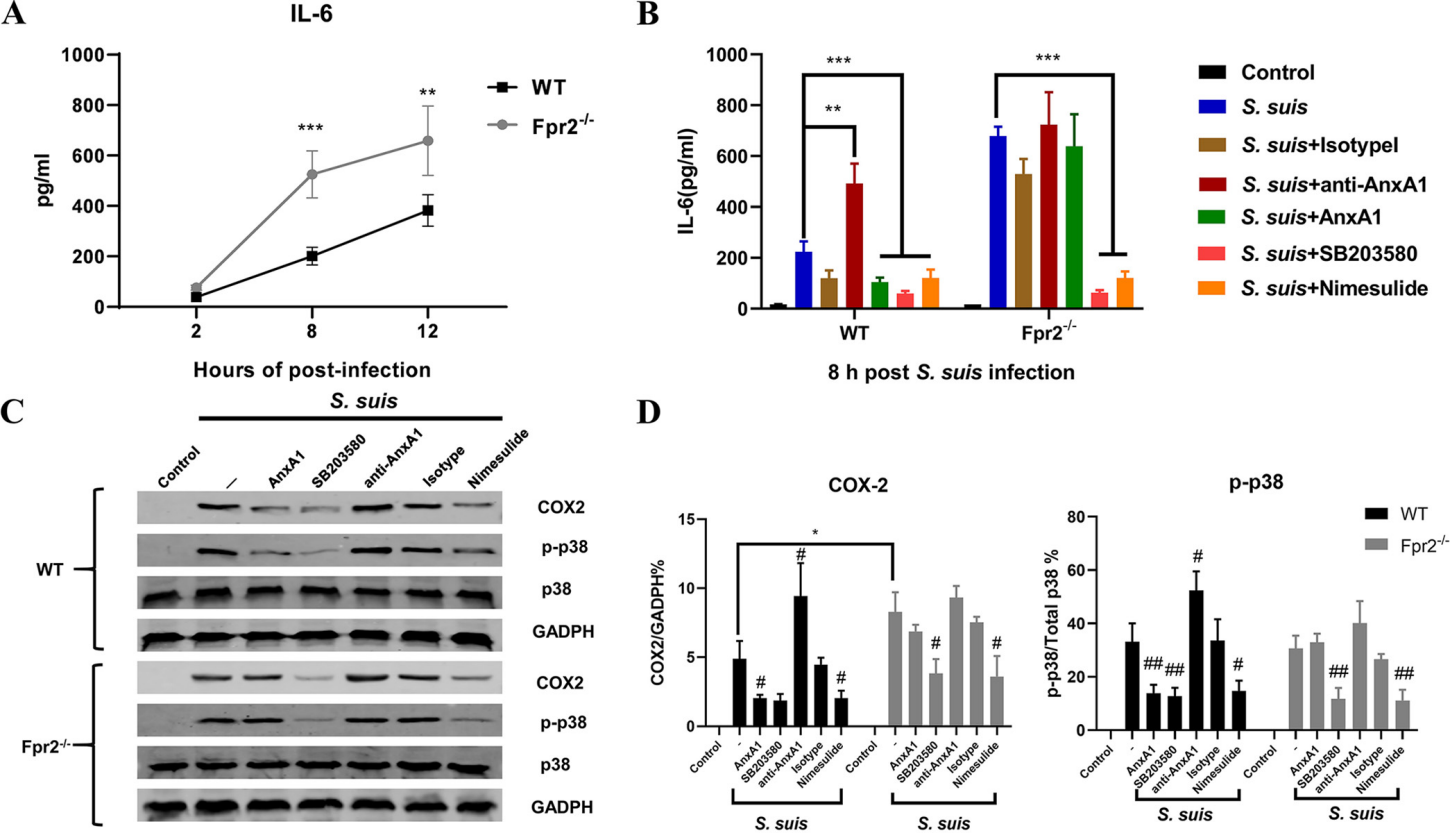
The IL-6-inhibitory function of AnxA1 was mediated by the Fpr2/p38/COX-2 pathway. (A) BMDMs from wild-type (WT) and Fpr2^−/−^ mice were infected with S. suis (initial MOI of 100), and the supernatants of the cell cultures were collected to measure IL-6 levels via ELISA at the indicated time points. (B) BMDMs were treated with AnxA1, SB203580, nimesulide, anti-AnxA1 antibody, or isotype antibody before S. suis (initial MOI of 100) infection, and IL-6 levels were measured at 8 h. (C) The cellular proteins were subjected to Western blotting to assess COX-2, p-p38, p38, and GADPH expression. (D) Quantitative analyses of COX-2 and p-p38 expression. #/*, *P* < 0.05, ##/**, *P* < 0.01, and ***, *P* < 0.001, compared with the S. suis-infected group (#) in the same mouse strain or as indicated (*).

To explore the mechanism of the involvement of the AnxA1-Fpr2 pathway in S. suis meningitis, *in vitro* experiments were conducted. WT and Fpr2^−/−^ bone marrow-derived macrophages (BMDMs) were infected with S. suis (initial multiplicity of infection [MOI] of 100), and IL-6 levels in the supernatant were measured at different time points (2, 8, and 12 h). Inoculation of S. suis induced IL-6 expression in WT and Fpr2^−/−^ BMDMs, and IL-6 levels were significantly higher in Fpr2^−/−^ BMDM supernatant after 8 or 12 h of infection (Fig. 8A). Because other studies found that AnxA1 effectively reduces IL-6 production via effects on p38 mitogen-activated protein kinase (MAPK) and COX-2 signaling pathway (28, 29), we used SB203580 and nimesulide, which inhibit the ability of activated p38 to phosphorylate downstream substrates and COX-2 expression, respectively, to study the special molecular mechanisms involved in IL-6 protein expression. IL-6 production resulting from bacterial stimulation was reversed by AnxA1 treatment only in WT cells at 8 h after infection, and inhibition of phosphoryl p38 (p-p38) or COX-2 significantly inhibited S. suis-induced IL-6 expression irrespective of genotype, which indicates that the infection-induced increase of IL-6 production by these cells was dependent on p38 and COX-2 (Fig. 8B). AnxA1 treatment effectively attenuated the induction of p-p38 and COX-2 in WT cells, whereas these effects were abrogated in Fpr2^−/−^ cells (Fig. 8C and D). Anti-AnxA1 antibody, which strongly blocks endogenous AnxA1, significantly increased IL-6 secretion and COX-2/p38 expression compared with the effects of the isotype control in WT cells, which indicates that endogenous AnxA1 might share the same signaling pathway as exogenous AnxA1 (Fig. 8B to D). These results demonstrated that AnxA1 conferred a potent inhibitory effect on IL-6 production through the Fpr2/p38/COX-2 pathway.

## DISCUSSION

In this study, we uncovered that the lack of Fpr2 exacerbated S. suis-induced meningitis in mice infected with the Chinese virulent strain 05ZYH33, and AnxA1 administration exerted anti-inflammatory effects through Fpr2 in S. suis-infected mice. Our results suggested that Fpr2 negatively regulates neutrophil migration and cytokine induction during S. suis infection, which was found to be associated with decreased bacterial loads, increased survival, and alleviation of pathological injury. Meanwhile, exogenous AnxA1 additionally alleviated inflammatory responses, as characterized by reduced bacterial loads and cytokine levels, migratory astrocyte and microglial responses, and granulocyte invasion, during S. suis meningitis in WT but not Fpr2^−/−^ mice. We also found that AnxA1 decreased neutrophil adhesion through Fpr2, and AnxA1 decreased IL-6 expression through the Fpr2/p38/COX-2 pathway, which may clarify the mechanism by which the protein decreased cytokine expression and brain damage during S. suis meningitis.

The results of this study provide new insights into S. suis meningitis and uncover a distinct contribution of neutrophil recruitment to S. suis meningitis. Neutrophils are key regulatory cells of host defenses that are recruited to the CNS in great numbers during acute bacterial meningitis (21). It is known that the chromosomal pathogenicity island (PAI) designated SsPI-1 in Chinese epidemic S. suis strains is associated with human meningitis (30). The S. suis 05ZYH33 strain we used in this study is a highly virulent Chinese isolate that harbors the unique PAI designated 89K (renamed SsPI-1). Meanwhile, recent research found that NETs in the CNS hinder bacterial eradication during pneumococcal meningitis (31). NET components such as antimicrobial proteins and extracellular DNA have proinflammatory effects and contribute to disease severity (31).

As a phospholipid-binding protein, AnxA1 controls both the innate and adaptive immune responses by inhibiting the adhesion and transmigration of leukocytes. Machado et al. reported the nonredundant role of AnxA1 and its receptor Fpr2 in regulating the bacterial proliferation and inflammatory reaction during Streptococcus pneumoniae infection (20). The AnxA1-Fpr2 system also has a crucial function in hindering the resolution of cerebral inflammation in lipopolysaccharide (LPS)-induced sepsis (18). AnxA1 or its bioactive peptides potently inhibit neutrophil trafficking in mice, and this function was associated with the detachment of adherent neutrophils from the vascular wall (32, 33). Because our results align with prior findings, we speculate that endogenous AnxA1 is externalized on the surfaces of adherent neutrophils, thereby promoting detachment. The antimigratory action of AnxA1 was largely attenuated in Fpr2^−/−^ mice, which suggests that its effect on neutrophil recruitment depends mainly on Fpr2. Another reason for the lower neutrophil counts in the brains of AnxA1-treated WT mice with meningitis might be the induction of phagocytosis by AnxA1 in the periphery (34). Besides, we found that AnxA1 treatment only affected the bacterial burden in the brain, which suggests that AnxA1 probably controls the CNS-intrinsic inflammatory reaction by regulating glial reactivity. Future studies are needed to address whether neutrophil phagocytosis by astrocytes or microglia occurs during S. suis meningitis and whether such a potentially protective effect is mediated by Fpr2.

The primary immune effector cells of the innate immune response within the CNS are microglia and astrocytes, which respond to immunological stimuli and secrete proinflammatory mediators to activate other immune cells. Excessive inflammation can favor the multiplication of bacteria by exposing adhesion sites for bacteria on the injured area, which leads to dysfunctional leukocyte responses (35). IL-6 is considered an early response proinflammatory mediator that is upregulated during bacterial meningitis (36). The rapid secretion and metabolism of IL-6 are intensively correlated with acute neutrophilia in the infected area, which suggests a distinct role for activated neutrophils in controlling cytokine induction in S. suis-induced infection (37, 38). Our results revealed the primary local proinflammatory function of IL-6 during S. suis meningitis, which is modulated by the AnxA1-Fpr2 system. These results were consistent with previous findings of high IL-6 secretion in AnxA1^−/−^ lung fibroblasts following IL-1β stimulation and in AnxA1^−/−^ mice upon LPS administration (28, 39).

COX-2 and p38 MAPK have been identified as detrimental factors in intracerebral hemorrhage that contribute to inflammatory responses and blood-brain barrier disruption (20, 40). The key role of the p38 MAPK pathway in the regulation of COX-2 expression has been thoroughly studied by previous investigators (32). Although p38 MAPK and COX-2 upregulation by S. suis has previously been reported, COX-2 mRNA expression was downregulated after blocking MAPK signaling pathways in streptococcal cytolysin-stimulated macrophages (41, 42). This is the first study to demonstrate that p38/COX-2 expression is regulated by the AnxA1-Fpr2 system during S. suis infection. Our results are also consistent with previous findings of increased COX-2 expression in AnxA1^−/−^ mice (43). In addition, S. pneumoniae-induced p38 MAPK- and NF-κB-dependent COX-2 expression has been found in human lung epithelium (34). Although the role of NF-κB in proinflammatory signals has also been generally studied in the Fpr2 pathway, we found no significant difference in levels of NF-κB activation between AnxA1-treated WT and Fpr2^−/−^ S. suis-infected mice (data not shown).

In conclusion, we have described the functions of Fpr2 as a crucial regulator of the immune reaction in the S. suis infection murine model. Therapeutic intervention based on Fpr2 activation or AnxA1 administration should be considered for the treatment of S. suis meningitis. Additional clinical research is required to estimate the effect of AnxA1 or AnxA1-based mimetics as potential treatments for S. suis meningitis in humans.

## MATERIALS AND METHODS

### Ethics statement.

This research was conducted in compliance with the guidelines for laboratory animal care approved in China. All experimental procedures were approved by the Animal Ethics Committee of the Academy of Military Medical Sciences.

### Bacterial strains.

The virulent S. suis strain 05ZYH33 is a clinical isolate that was obtained originally from a patient with streptococcal toxic shock-like syndrome in Sichuan Province, China (44). The bacteria were grown overnight on Columbia agar (include 5% sheep blood) at 37°C in an atmosphere containing 5% CO_2_. Todd-Hewitt broth (THB) was used to inoculate the isolated colonies, and the bacteria were cultured at 37°C in a 5% CO_2_ incubator for 6 h.

### Generation of Fpr2-deficient mice.

Fpr2-deficient (Ensembl accession no. ENSMUSG00000052270) mice were generated using the Cre/LoxP system. Mice harboring a floxed allele of Fpr2 (*Fpr2^loxP/^*^+^) were generated by Cyagen Biosciences (Guangzhou, China), and *Fpr2^loxP^*^/+^ mice were interbred to generate *Fpr2^loxP/loxP^* mice. *Fpr2^loxP/loxP^* mice were then mated with EIIa-cre transgenic mice (expressing Cre recombinase in nearly all tissues) to generate a null allele of Fpr2 (Fpr2^+/−^). *Fpr2*^+/−^ mice were identified by PCR genotyping using multiple primers (mFpr2_F1 [CTCATACGCATTTGCTGTCTTCACAC], mFpr2_R1 [TCCAATTATATCCCTTTCATGGCAAAC], and mFpr2_F3 [ACAAGGGCCTGCATGTGCCCTCTG]). Finally, Fpr2^+/−^ mice were interbred to generate Fpr2^−/−^ mice.

### Murine model.

S. suis infection in C57BL/6 mice was performed as previously described with some modifications (2). C57BL/6 mice (6 to 8 weeks old) were anesthetized with pentobarbital sodium (50 mg/kg) and inoculated by injecting 10 μl of S. suis suspension (1.25 × 10^5^ CFU) into the cisterna magna to evaluate the role of Fpr2 during S. suis meningitis. Mock-infected mice were injected with 10 μl of THB, and Fpr2 mRNA transcription levels in the brain were evaluated. In the intervention experiment, BOC-2 (600 ng/kg) or vehicle (phosphate-buffered saline [PBS]) was injected intraperitoneally 1 h before infection with S. suis. The infection was also executed in WT and Fpr2^−/−^ mice to directly evaluate the effect of Fpr2 on S. suis meningitis development. After WT and Fpr2^−/−^ mice were intracisternally infected with S. suis (1.25 × 10^5^ CFU), mortality, bacterial loads, and histopathological changes were monitored after 14 h. Brain tissue was collected to evaluate cytokine transcription, MPO expression, and neutrophil recruitment at 14 h. For the AnxA1 intervention experiment, 25 or 50 µg/kg recombinant AnxA1 protein (Biorbyt) was administered 10 min before S. suis infection via i.v. injection. The blood bacterial load of infected mice was evaluated by collecting 10 µl of blood from the caudal vein, and the bacterial loads in various tissues (liver, spleen, brain, and kidney) were evaluated by collecting 100 µl of homogenate from homogenized tissues, followed by appropriate dilution and plating on Todd-Hewitt agar.

### Quantitative real-time PCR.

Brain tissue from WT or Fpr2^−/−^ mice was dissected and snap-frozen in liquid nitrogen. RNA was extracted and quantified by spectrophotometry. The ThermoScript RT-PCR System (Invitrogen) was used to amplify cDNA, and then the products were used immediately for SYBR green reverse transcription-PCR (RT-PCR). The expression of the target gene was monitored using the StepOnePlus apparatus (Applied Biosystems) in compliance with the manufacturer’s recommendations. Relative quantification was calculated using the threshold cycle (△*C_T_*) method, which produces ratios of expression between target genes and the housekeeping reference gene coding for GADPH (glyceraldehyde-3-phosphate dehydrogenase). The primers for Fpr2, CXCL2, CXCL1, IL-6, IL-1β, TNF-α, IFN-γ, and GADPH are listed in Table S1 in the supplemental material.

### MPO.

Myeloperoxidase (MPO) protein levels in cell-free tissue were determined using an MPO colorimetric activity assay kit (Njjcbio, China) in compliance with the manufacturer’s protocol.

### FACS analysis.

Mice were anesthetized at the indicated time points and perfused with PBS transcardially. Brains were collected and mashed onto a 70-μm-pore strainer. DNase I (200 μg/ml) and collagenase (0.5 mg/ml) were used to digest brain suspension for 20 min at room temperature. Cells were spun down, resuspended in 7 ml of 30% Percoll in PBS containing 5% fetal calf serum (FCS), and then centrifuged at 1,400 × *g* for 20 min at 4°C. After the upper Percoll layers were discarded, the cell pellet was resuspended in PBS–3% FCS buffer. Contaminating erythrocytes were lysed with ACK lysis buffer for 3 min. After washing, a few cells were resuspended and stained with trypan blue to assess cell counts and viability by hemocytometer. For antibody labeling, the cell suspension was incubated with Mouse Fc Block (BioLegend) for 15 min at 4°C, followed by incubation with a mixture of fluorescent antibodies in PBS–3% FCS buffer for 25 min at 4°C. The following antibodies were used: fluorescein isothiocyanate (FITC)-conjugated anti-mouse CD45 (30-F11; BD Pharmingen), phycoerythrin (PE)-conjugated anti-mouse Ly6G (1A8; BD Pharmingen), peridinin chlorophyll protein (PerCP)-Cy5.5-conjugated anti-mouse CD11b (M1/70; Biolegend), and allophycocyanin (APC)-Cy7-conjugated anti-mouse fixable viability dye eFluor780 (eBioscience). After staining, the fluorescence-activated cell sorter (FACS) analysis was performed with a BD FACSVerse flow cytometer. Data analysis was performed by FlowJo software.

### Scoring.

Clinical scoring was performed in accordance with a formerly developed scoring list for a bacterial meningitis mouse model (see Table S2 in the supplemental material) (45). Animals reaching humane endpoint criteria (a total score of >15) were humanely euthanized.

### Cytokine measurement.

For proinflammatory mediator evaluation in the brain, ice-cold saline solution, which included protease inhibitor and 0.4% CHAPS {3-[(3-cholamidopropyl)-dimethylammonio]-1-propanesulfonate}, was added to frozen brains, which were then homogenized using a tissue homogenizer and centrifuged at 10,000 × *g* for 10 min at 4°C to collect supernatants of brain homogenate. CXCL1, CXCL2, IL-6, and IL-1β levels were measured by ELISA (Neobioscience, China) according to the manufacturer’s recommendations, as described previously (2).

### Neutrophil adhesion experiment.

Neutrophils were isolated from the bone marrow of naive mice using Histopaque separation medium via a density gradient centrifugation method (46). Cell purity was confirmed by flow cytometry (>90% Ly6G^+^). bEnd.3 cells were purchased from ATCC (no. CRL-2299) and maintained in Dulbecco’s modified Eagle’s medium (DMEM) containing 10% fetal bovine serum (FBS), l-glutamine, and penicillin-streptomycin (5,000 U/ml). Cells were cultured in a petri dish and 24-well tissue culture plates at 37°C with 5% CO_2_. For adhesion experiments, neutrophils were stained with 1,1′-dioctadecyl-3,3,3′,3′-tetramethylindocarbocyanine for 20 min in RPMI 1640 (0.1% FBS) (47). After treatment with AnxA1 or vehicle, bEnd.3 cells were incubated with heat-killed S. suis (HkSs) (MOI of 100) for 6 h, and fragments of bacteria removed by washing with RPMI. The labeled neutrophils (1 × 10^5^ cells/ml) were then incubated with bEnd.3 cells for 30 min. Nonadherent neutrophils were washed away by RPMI gently. Adherent neutrophils on bEnd.3 cells were counted in five randomly selected areas under a motorized inverted microscope.

### Western blotting.

The protein content of the lysate was measured via the bicinchoninic acid (BCA) protein assay. Cells lysates were boiled in protein loading buffer, analyzed by SDS-PAGE, and electrophoretically transferred to polyvinylidene fluoride membranes. The membranes were blocked with TBST (Tris-buffered saline with Tween 20) containing 5% bovine serum albumin (BSA) and incubated with primary antibodies overnight at 4°C. Immunodetection of target proteins was conducted with specific antibodies against p38 (Cell Signaling Technology), p-p38 (Cell Signaling Technology), COX-2 (Proteintech), AnxA1 (Proteintech), and GADPH (Proteintech). The blots were incubated with the appropriate secondary antibody diluted in TBST for 1 h. Immunoblots were detected via immunofluorescence.

### Histopathological studies.

Mice were euthanized, and liver, lung, brain, and spleen tissue were recovered and fixed in 10% buffered formalin. These tissues were stained with hematoxylin and eosin and examined via Olympus BX53 microscopy.

### Immunohistochemistry.

The sections from paraffin-embedded brain were used to label target antibody, and the experiment was conducted as previously described (48). Sections were deparaffinized and pretreated for 10 min by an autoclave in Tris-EDTA buffer (pH 9.0) before undergoing blocking with 5% normal goat serum. The sections were washed in PBS and incubated with anti-GFAP (Proteintech) or anti-Iba-1 (Proteintech) overnight at 4°C. On the next day, the sections were washed with PBS, and then the slides were incubated with the appropriate secondary antibody for 1 h at room temperature. After being washed with PBS adequately, the slides were counterstained with hematoxylin and mounted with Aquatex. Pictures were captured using Olympus BX53 microscope and analyzed using ImagePro-Plus software.

### Generation of BMDMs.

The tibias and femurs of WT or Fpr2^−/−^ mice were used to prepare BMDMs using protocols modified from Lutz et al. (49). Bone marrow-derived cells were prepared by flushing tibias and femurs, and cells were grown in DMEM containing 10% FBS. Cells were cultured for 7 days with 100 ng/ml M-CSF (on days 2 and 4, medium was replaced) in a humidified atmosphere (37°C, 5% CO_2_) in the presence of penicillin-streptomycin (50 UI/ml). On day 6 of culture, cells were plated and stimulated with S. suis and/or reagents after 24 h.

### Stimulation of BMDMs with bacteria.

WT and Fpr2^−/−^ BMDMs were stimulated with S. suis (initial MOI of 100), and the supernatants were collected to detect IL-6 levels at the indicted time points. In a separate experiment, WT and Fpr2^−/−^ BMDMs were pretreated with AnxA1 protein, 5 μM SB203580 (an inhibitor of p38 MAPK; MedChemExpress), 10 nM nimesulide (an inhibitor of COX-2; MedChemExpress), anti-AnxA1 antibody (1:200), or isotype control before S. suis infection (initial MOI of 100). Then, the cultural supernatants were collection to detect IL-6 levels, and cells were lysed to detect the expression of the indicated protein by Western blotting after 8 h.

### Statistical analysis.

Bacterial loads and brain-invading neutrophil counts were analyzed using the Mann-Whitney *U* test. Survival experiments were analyzed using the Kaplan-Meier method and log rank test. Significance between two independent groups was determined via two-way analysis of variance followed by Bonferroni’s multiple-comparison test. *P* < 0.05 was defined statistical significance. GraphPad 8.0 software was used for analysis.

## Supplementary Material

Supplemental file 1

## References

[1] Wang J, Kong D, Zhang S, Jiang H, Zheng Y, Zang Y, Hao H, Jiang Y. 2015. Interaction of fibrinogen and muramidase-released protein promotes the development of *Streptococcus suis* meningitis. Front Microbiol 6:1001. doi:10.3389/fmicb.2015.01001. 26441928 PMC4585153

[2] Auger JP, Benoit-Biancamano MO, Bedard C, Segura M, Gottschalk M. 2019. Differential role of MyD88 signaling in *Streptococcus suis* serotype 2-induced systemic and central nervous system diseases. Int Immunol 31:697–714. doi:10.1093/intimm/dxz033. 30944920

[3] Hlebowicz M, Jakubowski P, Smiatacz T. 2019. *Streptococcus suis* meningitis: epidemiology, clinical presentation and treatment. Vector Borne Zoonotic Dis 19:557–562. doi:10.1089/vbz.2018.2399. 30855223

[4] Geng H, Zhu L, Yuan Y, Zhang W, Li W, Wang J, Zheng Y, Wei K, Cao W, Wang H, Jiang Y. 2008. Identification and characterization of novel immunogenic proteins of *Streptococcus suis* serotype 2. J Proteome Res 7:4132–4142. doi:10.1021/pr800196v. 18630869

[5] Lachance C, Gottschalk M, Gerber PP, Lemire P, Xu J, Segura M. 2013. Exacerbated type II interferon response drives hypervirulence and toxic shock by an emergent epidemic strain of *Streptococcus suis*. Infect Immun 81:1928–1939. doi:10.1128/IAI.01317-12. 23509145 PMC3676015

[6] Ma F, Chang X, Wang G, Zhou H, Ma Z, Lin H, Fan H. 2018. *Streptococcus suis* serotype 2 stimulates neutrophil extracellular traps formation via activation of p38 MAPK and ERK1/2. Front Immunol 9:2854. doi:10.3389/fimmu.2018.02854. 30581435 PMC6292872

[7] Buckingham JC, John CD, Solito E, Tierney T, Flower RJ, Christian H, Morris J. 2006. Annexin 1, glucocorticoids, and the neuroendocrine-immune interface. Ann N Y Acad Sci 1088:396–409. doi:10.1196/annals.1366.002. 17192583 PMC1855441

[8] Renshaw D, Montero-Melendez T, Dalli J, Kamal A, Brancaleone V, D'Acquisto F, Cirino G, Perretti M. 2010. Downstream gene activation of the receptor ALX by the agonist annexin A1. PLoS One 5:e12771. doi:10.1371/journal.pone.0012771. 20862244 PMC2941452

[9] Sugimoto MA, Vago JP, Teixeira MM, Sousa LP. 2016. Annexin A1 and the resolution of inflammation: modulation of neutrophil recruitment, apoptosis, and clearance. J Immunol Res 2016:8239258. doi:10.1155/2016/8239258. 26885535 PMC4738713

[10] Perretti M, Flower RJ. 2004. Annexin 1 and the biology of the neutrophil. J Leukoc Biol 76:25–29. doi:10.1189/jlb.1103552. 14966195

[11] Corminboeuf O, Leroy X. 2015. FPR2/ALXR agonists and the resolution of inflammation. J Med Chem 58:537–559. doi:10.1021/jm501051x. 25365541

[12] Polli-Lopes AC, Estofolete CF, Oliani SM, Zucoloto S, Cunha FQ, Gil CD. 2013. Myenteric denervation in gastric carcinogenesis: differential modulation of nitric oxide and annexin-A1. Int J Clin Exp Pathol 6:13–23. 23236538 PMC3515986

[13] Ding Y, Flores J, Klebe D, Li P, McBride DW, Tang J, Zhang JH. 2020. Annexin A1 attenuates neuroinflammation through FPR2/p38/COX-2 pathway after intracerebral hemorrhage in male mice. J Neurosci Res 98:168–178. doi:10.1002/jnr.24478. 31157469 PMC6854313

[14] Hayhoe RP, Kamal AM, Solito E, Flower RJ, Cooper D, Perretti M. 2006. Annexin 1 and its bioactive peptide inhibit neutrophil-endothelium interactions under flow: indication of distinct receptor involvement. Blood 107:2123–2130. doi:10.1182/blood-2005-08-3099. 16278303

[15] Bena S, Brancaleone V, Wang JM, Perretti M, Flower RJ. 2012. Annexin A1 interaction with the FPR2/ALX receptor: identification of distinct domains and downstream associated signaling. J Biol Chem 287:24690–24697. doi:10.1074/jbc.M112.377101. 22610094 PMC3397896

[16] Ye RD, Boulay F, Wang JM, Dahlgren C, Gerard C, Parmentier M, Serhan CN, Murphy PM. 2009. International Union of Basic and Clinical Pharmacology. LXXIII. Nomenclature for the formyl peptide receptor (FPR) family. Pharmacol Rev 61:119–161. doi:10.1124/pr.109.001578. 19498085 PMC2745437

[17] Sahagun-Ruiz A, Colla JS, Juhn J, Gao JL, Murphy PM, McDermott DH. 2001. Contrasting evolution of the human leukocyte N-formylpeptide receptor subtypes FPR and FPRL1R. Genes Immun 2:335–342. doi:10.1038/sj.gene.6363787. 11607790

[18] Gavins FN, Hughes EL, Buss NA, Holloway PM, Getting SJ, Buckingham JC. 2012. Leukocyte recruitment in the brain in sepsis: involvement of the annexin 1-FPR2/ALX anti-inflammatory system. FASEB J 26:4977–4989. doi:10.1096/fj.12-205971. 22964301

[19] Spurr L, Nadkarni S, Pederzoli-Ribeil M, Goulding NJ, Perretti M, D'Acquisto F. 2011. Comparative analysis of annexin A1-formyl peptide receptor 2/ALX expression in human leukocyte subsets. Int Immunopharmacol 11:55–66. doi:10.1016/j.intimp.2010.10.006. 20974309

[20] Machado MG, Tavares LP, Souza GVS, Queiroz-Junior CM, Ascencao FR, Lopes ME, Garcia CC, Menezes GB, Perretti M, Russo RC, Teixeira MM, Sousa LP. 2020. The annexin A1/FPR2 pathway controls the inflammatory response and bacterial dissemination in experimental pneumococcal pneumonia. FASEB J 34:2749–2764. doi:10.1096/fj.201902172R. 31908042

[21] Auger JP, Rivest S, Benoit-Biancamano MO, Segura M, Gottschalk M. 2019. Inflammatory monocytes and neutrophils regulate *Streptococcus suis*-induced systemic inflammation and disease but are not critical for the development of central nervous system disease in a mouse model of infection. Infect Immun 88:e00787-19. doi:10.1128/IAI.00787-19. PMC7035915 31818962

[22] Solito E, McArthur S, Christian H, Gavins F, Buckingham JC, Gillies GE. 2008. Annexin A1 in the brain—undiscovered roles? Trends Pharmacol Sci 29:135–142. doi:10.1016/j.tips.2007.12.003. 18262660

[23] Senchenkova EY, Ansari J, Becker F, Vital SA, Al-Yafeai Z, Sparkenbaugh EM, Pawlinski R, Stokes KY, Carroll JL, Dragoi AM, Qin CX, Ritchie RH, Sun H, Cuellar-Saenz HH, Rubinstein MR, Han YW, Orr AW, Perretti M, Granger DN, Gavins FNE. 2019. Novel role for the AnxA1-Fpr2/ALX signaling axis as a key regulator of platelet function to promote resolution of inflammation. Circulation 140:319–335. doi:10.1161/CIRCULATIONAHA.118.039345. 31154815 PMC6687438

[24] Auger JP, Fittipaldi N, Benoit-Biancamano MO, Segura M, Gottschalk M. 2016. Virulence studies of different sequence types and geographical origins of *Streptococcus suis* serotype 2 in a mouse model of infection. Pathogens 5:48. doi:10.3390/pathogens5030048. PMC5039428 27409640

[25] Ransohoff RM, Brown MA. 2012. Innate immunity in the central nervous system. J Clin Invest 122:1164–1171. doi:10.1172/JCI58644. 22466658 PMC3314450

[26] Oldekamp S, Pscheidl S, Kress E, Soehnlein O, Jansen S, Pufe T, Wang JM, Tauber SC, Brandenburg LO. 2014. Lack of formyl peptide receptor 1 and 2 leads to more severe inflammation and higher mortality in mice with of pneumococcal meningitis. Immunology 143:447–461. doi:10.1111/imm.12324. 24863484 PMC4212958

[27] Lim LH, Solito E, Russo-Marie F, Flower RJ, Perretti M. 1998. Promoting detachment of neutrophils adherent to murine postcapillary venules to control inflammation: effect of lipocortin 1. Proc Natl Acad Sci U S A 95:14535–14539. doi:10.1073/pnas.95.24.14535. 9826735 PMC24408

[28] Yang YH, Aeberli D, Dacumos A, Xue JR, Morand EF. 2009. Annexin-1 regulates macrophage IL-6 and TNF via glucocorticoid-induced leucine zipper. J Immunol 183:1435–1445. doi:10.4049/jimmunol.0804000. 19553536

[29] Zhao Y, Usatyuk PV, Gorshkova IA, He D, Wang T, Moreno-Vinasco L, Geyh AS, Breysse PN, Samet JM, Spannhake EW, Garcia JG, Natarajan V. 2009. Regulation of COX-2 expression and IL-6 release by particulate matter in airway epithelial cells. Am J Respir Cell Mol Biol 40:19–30. doi:10.1165/rcmb.2008-0105OC. 18617679 PMC5459547

[30] Kerdsin A, Dejsirilert S, Puangpatra P, Sripakdee S, Chumla K, Boonkerd N, Polwichai P, Tanimura S, Takeuchi D, Nakayama T, Nakamura S, Akeda Y, Gottschalk M, Sawanpanyalert P, Oishi K. 2011. Genotypic profile of *Streptococcus suis* serotype 2 and clinical features of infection in humans, Thailand. Emerg Infect Dis 17:835–842. doi:10.3201/eid1705.100754. 21529392 PMC3321758

[31] Mohanty T, Fisher J, Bakochi A, Neumann A, Cardoso JFP, Karlsson CAQ, Pavan C, Lundgaard I, Nilson B, Reinstrup P, Bonnevier J, Cederberg D, Malmstrom J, Bentzer P, Linder A. 2019. Neutrophil extracellular traps in the central nervous system hinder bacterial clearance during pneumococcal meningitis. Nat Commun 10:1667. doi:10.1038/s41467-019-09040-0. 30971685 PMC6458182

[32] Gavins FN, Hickey MJ. 2012. Annexin A1 and the regulation of innate and adaptive immunity. Front Immunol 3:354. doi:10.3389/fimmu.2012.00354. 23230437 PMC3515881

[33] Liu NK, Zhang YP, Han S, Pei J, Xu LY, Lu PH, Shields CB, Xu XM. 2007. Annexin A1 reduces inflammatory reaction and tissue damage through inhibition of phospholipase A2 activation in adult rats following spinal cord injury. J Neuropathol Exp Neurol 66:932–943. doi:10.1097/nen.0b013e3181567d59. 17917587

[34] N'Guessan PD, Hippenstiel S, Etouem MO, Zahlten J, Beermann W, Lindner D, Opitz B, Witzenrath M, Rosseau S, Suttorp N, Schmeck B. 2006. *Streptococcus pneumoniae* induced p38 MAPK- and NF-kappaB-dependent COX-2 expression in human lung epithelium. Am J Physiol Lung Cell Mol Physiol 290:L1131–L1138. doi:10.1152/ajplung.00383.2005. 16414978

[35] Abreu MT, Fukata M, Arditi M. 2005. TLR signaling in the gut in health and disease. J Immunol 174:4453–4460. doi:10.4049/jimmunol.174.8.4453. 15814663

[36] Jones SA. 2005. Directing transition from innate to acquired immunity: defining a role for IL-6. J Immunol 175:3463–3468. doi:10.4049/jimmunol.175.6.3463. 16148087

[37] Dalrymple SA, Lucian LA, Slattery R, McNeil T, Aud DM, Fuchino S, Lee F, Murray R. 1995. Interleukin-6-deficient mice are highly susceptible to Listeria monocytogenes infection: correlation with inefficient neutrophilia. Infect Immun 63: 2262–2268. doi:10.1128/IAI.63.6.2262-2268.1995. 7768607 PMC173295

[38] Cole N, Krockenberger M, Bao S, Beagley KW, Husband AJ, Willcox M. 2001. Effects of exogenous interleukin-6 during *Pseudomonas aeruginosa* corneal infection. Infect Immun 69:4116–4119. doi:10.1128/IAI.69.6.4116-4119.2001. 11349084 PMC98477

[39] Yang YH, Toh ML, Clyne CD, Leech M, Aeberli D, Xue J, Dacumos A, Sharma L, Morand EF. 2006. Annexin 1 negatively regulates IL-6 expression via effects on p38 MAPK and MAPK phosphatase-1. J Immunol 177:8148–8153. doi:10.4049/jimmunol.177.11.8148. 17114490

[40] Candelario-Jalil E, González-Falcón A, García-Cabrera M, León OS, Fiebich BL. 2007. Post-ischaemic treatment with the cyclooxygenase-2 inhibitor nimesulide reduces blood-brain barrier disruption and leukocyte infiltration following transient focal cerebral ischaemia in rats. J Neurochem 100:1108–1120. doi:10.1111/j.1471-4159.2006.04280.x. 17176264

[41] Lachance C, Segura M, Dominguez-Punaro MC, Wojewodka G, De Sanctis JB, Radzioch D, Gottschalk M. 2014. Deregulated balance of omega-6 and omega-3 polyunsaturated fatty acids following infection by the zoonotic pathogen *Streptococcus suis*. Infect Immun 82:1778–1785. doi:10.1128/IAI.01524-13. 24549326 PMC3993453

[42] Blaschke U, Beineke A, Klemens J, Medina E, Goldmann O. 2017. Induction of cyclooxygenase 2 by *Streptococcus pyogenes* is mediated by cytolysins. J Innate Immun 9:587–597. doi:10.1159/000479153. 28813715

[43] Roviezzo F, Getting SJ, Paul-Clark MJ, Yona S, Gavins FNE, Perretti M, Hannon R, Croxtall JD, Buckingham JC, Flower RJ. 2002. The annexin-1 knockout mouse: what it tells us about the inflammatory response. J Physiol Pharmacol 53:541–553. 12516535

[44] Ye C, Zheng H, Zhang J, Jing H, Wang L, Xiong Y, Wang W, Zhou Z, Sun Q, Luo X, Du H, Gottschalk M, Xu J. 2009. Clinical, experimental, and genomic differences between intermediately pathogenic, highly pathogenic, and epidemic *Streptococcus suis*. J Infect Dis 199:97–107. doi:10.1086/594370. 19016627

[45] Mook-Kanamori B, Geldhoff M, Troost D, van der Poll T, van de Beek D. 2012. Characterization of a pneumococcal meningitis mouse model. BMC Infect Dis 12:71. doi:10.1186/1471-2334-12-71. 22455545 PMC3364848

[46] Swamydas M, Luo Y, Dorf ME, Lionakis MS. 2015. Isolation of mouse neutrophils. Curr Protoc Immunol 110:3.20.1–3.20.15. 26237011 10.1002/0471142735.im0320s110 PMC4574512

[47] Chen P-J, Wang Y-L, Kuo L-M, Lin C-F, Chen C-Y, Tsai Y-F, Shen J-J, Hwang T-L. 2016. Honokiol suppresses TNF-α-induced neutrophil adhesion on cerebral endothelial cells by disrupting polyubiquitination and degradation of IκBα. Sci Rep 6:26554. doi:10.1038/srep26554. 27212040 PMC4876378

[48] Liu Q, Chen Y, Shen C, Xiao Y, Wang Y, Liu Z, Liu X. 2017. Chicoric acid supplementation prevents systemic inflammation-induced memory impairment and amyloidogenesis via inhibition of NF-kappaB. FASEB J 31:1494–1507. doi:10.1096/fj.201601071R. 28003341

[49] Lutz MB, Kukutsch N, Ogilvie AL, Rössner S, Koch F, Romani N, Schuler G. 1999. An advanced culture method for generating large quantities of highly pure dendritic cells from mouse bone marrow. J Immunol Methods 223:77–92. doi:10.1016/S0022-1759(98)00204-X. 10037236

