# Supplementary material for: Annexin A1 Attenuates Neutrophil Migration and IL-6 Expression through Fpr2 in a Mouse Model of Streptococcus suis-Induced Meningitis
Source: Infect Immun. 2021 Feb 16;89(3):e00680-20. doi: 10.1128/IAI.00680-20 (PMC8097268; doi:10.1128/IAI.00680-20)
Supplement: Supplemental file 1 [file IAI.00680-20-s0001.pdf]

TABLE S1 Primer sequences for quantitative real-time PCR gene analysis.

Name	Sequence (5′→3′)
Fpr2_F	ACTGTGAGCCTGGCTAGGAA
Fpr2_R	CATCAGTTTGAGCCCAGGAT
CXCL1_F	CTTGCCTTGACCCTGAAGC
CXCL1_R	AGGTGCCATCAGAGCAGTCT
CXCL2_F	TCAAGAACATCCAGAGCTTGAG
CXCL2_R	TTCAGGGTCAAGGCAAACTT
IL-6_F	CCGGAGAGGAGACTTCACAG
IL-6_R	TCCACGATTTCCCAGAGAAC
IL-1β_F	GCAACTGTTCCTGAACTCAACT
IL-1β_R	ATCTTTTGGGGTCCGTCAACT
TNFα _F	CCCAGGTATATGGGCTCATACC
TNFα_R	GCCGATTTGCTATCTCATACCAGG
IFN-γ_F	TGAGCTCATTGAATGCTTGG
IFN-γ_R	ACAGCAAGGCGAAAAAGGAT
ICAM-1_F	CACACTTCACAGTTACTTGGCTCCC
ICAM-1_R	GCTGCGTTTTGGAGCTAGCGGACC
GAPDH_F	GTCTTCACCACCATGGAG
GAPDH_R	CCAAAGTTGTCATGGATGACC

TABLE S2 Clinical score parameters, assessed values and weighted scores

Parameter	Value	Weighted score	Maxium score
Weight loss from baseline	5%	0	
	10%	1	
	15%	2	
	20%	3	
	25%	4	4
Activity	normal	0	
	increased/decreased	1	
	mildely deminished	1	
	deminished	2	
	severely deminished	3	
	coma	4	4
Time to return to upright position	normal	0	
	upright < 5 sec	2	
	upright < 30 sec	4	
	no turn upright	6	6
Coat	normal	0	
	deminished grooming	1	
	soiled	1	
	piloerection	1	3
Posture	normal	0	
	slightly hunched back	1	
	severe hunched back	2	2
Eyes	normal	0	
	protruding	1	
	sunken eyes	1	
	closed eyelids	1	
	discharge	1	4
Respiration rate (per min)	> 150	0	
	< 150	1	
	< 100	2	
	< 75	3	
	< 50	4	4
Breathing	irregular	2	
	labored	2	4
Neurologic exam	normal	0	
	ataxia	2	
	limb paresis/paralysis	2	
	epileptic seizure	2	
	status epilepticus	6	10
Total			41

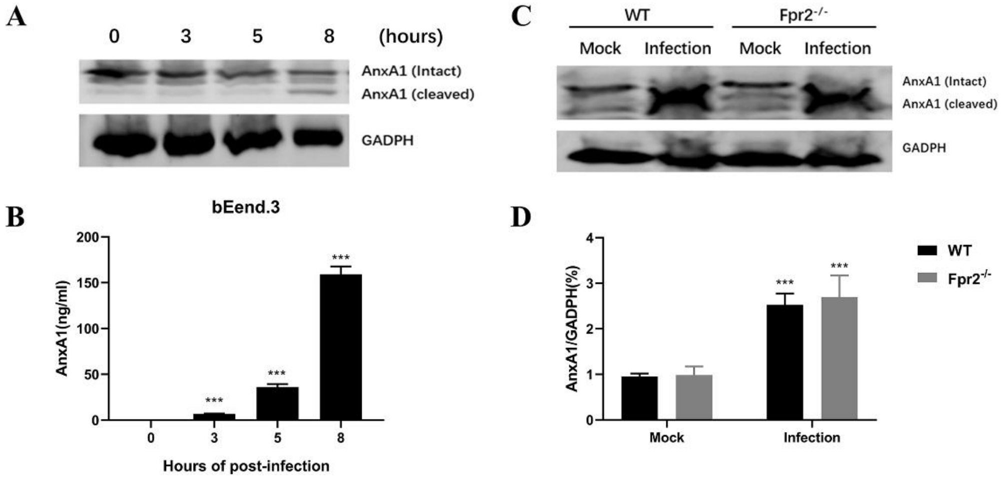

FIG S1 (A) The bEnd.3 cells were infected with *S. suis* (initial MOI =100) at the indicted times, the cellular AnxA1 expression was analyzed via western blotting, (B) and the cell supernatants of infected bEnd.3 cells were collected to detect AnxA1 expression via ELISA. (C, D) WT and Fpr2$^{-/-}$ mice were intracisternally inoculated with *S. suis* (1.25 × 10^5 CFU), the brain was subjected to western blot analysis at 14 h to assess endogenous AnxA1 expression. ***, *P* < 0.001 vs. 0 h (or mock) group in the same mouse strain.

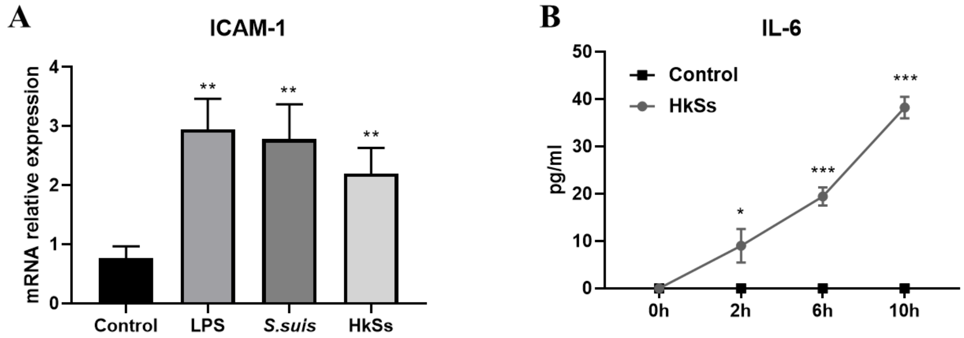

FIG S2 (A) The bEnd.3 cells were stimulated with LPS(10 ng/ml), *S. suis* (initial MOI = 50) or HkSs (MOI =100), then the transcription of ICAM-1 was analyzed at the 6 h via quantitative real-time PCR. (B) The bEnd.3 cells were stimulated with HkSs (MOI =100) at the indicted times and the cell supernatants were collected to detect IL-6 levels via ELISA. * $P < 0.05$ and ***, $P < 0.001$ compared with the Control group.
